# Association of Prenatal Ambient Air Pollution Exposure With Placental Mitochondrial DNA Copy Number, Telomere Length and Preeclampsia

**DOI:** 10.3389/ftox.2021.659407

**Published:** 2021-05-26

**Authors:** Yumjirmaa Mandakh, Anna Oudin, Lena Erlandsson, Christina Isaxon, Stefan R. Hansson, Karin Broberg, Ebba Malmqvist

**Affiliations:** ^1^Environment Society and Health, Division of Occupational and Environmental Medicine, Department of Laboratory Medicine, Lund University, Lund, Sweden; ^2^Section of Sustainable Health, Department of Public Health and Clinical Medicine, Umeå University, Umeå, Sweden; ^3^Division of Obstetrics and Gynecology, Department of Clinical Sciences Lund, Lund University, Lund, Sweden; ^4^Ergonomics and Aerosol Technology, Department of Design Sciences, Lund University, Lund, Sweden; ^5^Department of Obstetrics & Gynaecology, Skåne University Hospital, Malmö, Sweden; ^6^Division of Occupational and Environmental Medicine, Department of Laboratory Medicine, Lund University, Lund, Sweden

**Keywords:** preeclampsia, mitochondrial DNA copy number, telomere length, nitrogen oxides, placenta, ambient air pollution

## Abstract

**Background:** Studies have shown that ambient air pollution is linked to preeclampsia (PE), possibly via generation of oxidative stress in the placenta. Telomere length and mitochondrial DNA copy number (mtDNAcn) are sensitive to oxidative stress damage.

**Objective:** To study the association between prenatal exposure to ambient nitrogen oxides (NO_x_, a marker for traffic-related air pollution), and PE, as well as potential mediation effects by placental telomere length and mtDNAcn.

**Methods:** This is a cross-sectional study of 42 preeclamptic and 95 arbitrarily selected normotensive pregnant women with gestational ambient NO_x_ exposure assessment in southern Scania, Sweden. Hourly concentrations of NO_x_ were estimated at the residential addresses by a Gaussian-plume dispersion model with 100 × 100 m spatial resolutions and aggregated into trimester-specific mean concentrations. Placental relative mtDNAcn and telomere length were measured using qPCR. Linear and logistic regression models were used to investigate associations, adjusted for perinatal and seasonal characteristics.

**Results:** Exposure was categorized into low and high exposures by median cut-offs during first [11.9 μg/m^3^; interquartile range (IQR) 7.9, 17.9], second (11.6 μg/m^3^; IQR: 7.1, 21.1), third trimesters (11.9 μg/m^3^; IQR: 7.7, 19.5) and entire pregnancy (12.0 μg/m^3^; IQR: 7.6, 20.1). Increased risk of PE was found for high prenatal NO_x_ exposure during the first trimester (OR 4.0; 95% CI: 1.4, 11.1; *p* = 0.008), and entire pregnancy (OR 3.7; 95% CI: 1.3, 10.4; *p* = 0.012). High exposed group during the first trimester had lower placental relative mtDNAcn compared with low exposed group (−0.20; 95% CI: −0.36, −0.04; *p* = 0.01). Changes in relative mtDNAcn did not mediate the association between prenatal NO_x_ exposure and PE. No statistically significant association was found between placental relative telomere length, prenatal NO_x_ exposure and PE.

**Conclusion:** In this region with relatively low levels of air pollution, ambient NO_x_ exposure during the first trimester was associated with reduced placental relative mtDNAcn and an increased risk of PE. However, we did not find any evidence that mtDNAcn or TL mediated the association between air pollution and PE. Future research should further investigate the role of mtDNAcn for pregnancy complications in relation to exposure to ambient air pollution during pregnancy.

## Introduction

Ambient air pollution is a major global contributor to morbidity and mortality globally, accounting for 7.6% of all deaths worldwide in 2015 (Cohen et al., 2017). Exposure to ambient air pollution during pregnancy is particularly worrisome due to the health threat posed to both pregnant women and their fetuses. Several epidemiologic reviews have highlighted the possible association between maternal exposure to ambient air pollutants and pregnancy complications, such as gestational diabetes mellitus (Zhang et al., 2020), hypertensive disorders of pregnancy (Bai et al., 2020; Yu et al., 2020) and fetal growth restriction (FGR) (Fu et al., 2019). According to the Barker hypothesis, different environmental conditions present already at the time of conception may pose an increased risk of developing diseases later in life (Barker, 2004). Apparently, FGR associated with prenatal exposure to ambient air pollution appears to be a predisposing factor for impaired physical growth in the early years after birth (Clemente et al., 2017; Fossati et al., 2020).

The pathophysiological mechanisms linking ambient air pollution with pregnancy complications are not completely understood. One reason is the complex composition of air pollution containing a mixture of components, such as particulate matter (PM) of different sizes and compositions, ozone, nitrogen oxides (NO_x_), carbon monoxide and sulfur dioxide. Another reason is that pregnancy complications, especially preeclampsia (PE), tend to be caused by a dysfunctional placenta. According to the recommendation of International Society for the Study of Hypertension in Pregnancy (ISSHP), PE is defined as *de-novo* hypertension combined with maternal organ dysfunction and/*or utero-*placental dysfunction including FGR presenting after 20 weeks of gestation (Brown et al., 2018). More specifically, PE is more of a syndrome with different clinical manifestations than a well-defined disease (Gyselaers, 2020). It is classified into early (occurring before 34 gestational weeks) and late (occurring after 34 weeks) onset PE (Tranquilli et al., 2013).

The etiology and pathophysiology of early- and late-onset PE are still not fully understood, however, placental dysfunction due to syncytiotrophoblast stress may explain the underlying pathophysiologic mechanism of PE syndrome (Redman et al., 2021). Syncytiotrophoblast is the outer cell layer on placental villae in direct contact with maternal blood (Manna et al., 2019) and is formed by underlying cytotrophoblast fusion and senescence (Redman et al., 2021). The key characteristic of trophoblast senescence is a telomere shortening (Burton et al., 2017) and trophoblast fusion dependent on mitochondrial dynamics (Holland et al., 2017). Syncytiotrophoblast stress is suggested to be caused by insufficient placental perfusion and hypoxia due to incomplete placentation in early-onset PE or due to placental growth and maternal constitutional factors such as obesity, autoimmune disease and infection in late-onset PE (Redman et al., 2021).

Exposure to ambient air pollution is potentially associated with increased oxidative stress damage to nuclear and mitochondrial DNA (mtDNA), as well as lipid peroxidation, resulting in DNA mutations and cell death (Møller and Loft, 2010). Previous studies have reported a dose-dependent increase of a DNA adduct 8-hydroxy-deoxyguanosine in DNA isolated from lung, venous blood and urine from laboratory animals and humans exposed to diesel exhaust gases, such as NO_x_ and polycyclic aromatic hydrocarbons, and/or diesel exhaust particles, both the fine and ultrafine PM (Ichinose et al., 1997; Harri et al., 2005; Vinzents et al., 2005; Lee et al., 2012). Particularly, polycyclic aromatic hydrocarbons on ambient PM has been indicated to induce upregulation of cytochrome P450, which generates reactive oxygen species (ROS) (Bonvallot et al., 2001). Furthermore, recent evidence has suggested that elevated levels of malondialdehyde, a lipid peroxidation product of syncytiotrophoblast membranes, are linked to both short-term exposure to ambient air pollution (Li et al., 2020) and PE (Ramiro-Cortijo et al., 2020). Taken together, air pollution-induced oxidative stress may cause placental dysfunction (Saenen et al., 2019). Interestingly, telomeres and mitochondria are both considered to be important targets of oxidative stress induced by environmental pollutants (Martens and Nawrot, 2016).

Telomeres are specialized structures located at the end of each chromosome. In human somatic cells, telomeres gradually shorten during cell division until they reach a critical point that triggers replicative cell senescence or apoptosis (Hayflick and Moorhead, 1961; Victorelli and Passos, 2017; Pańczyszyn et al., 2020). The high guanine content in the telomeres render them susceptible to ROS and accelerated telomere length shortening has been found to be associated with oxidative stress (Richter and Zglinicki, 2007; Pańczyszyn et al., 2020). A growing body of literature suggests that exposure to environmental pollutants during pregnancy is associated with changes in placental telomere length (Lin et al., 2013; Bijnens et al., 2015; Herlin et al., 2019). Moreover, it has been indicated that placentas from pregnancies complicated with PE and FGR have shorter telomere length as compared with uncomplicated pregnancies (Biron-Shental et al., 2010).

Mitochondria are cell organelles that are responsible for ATP production and several metabolic pathways, such as the heme synthesis. Mitochondria in placental cytotrophoblast and syncytiotrophoblast have different structure and functions (Bustamante et al., 2014). Due to its location in the mitochondrial matrix and its structure without histones, mtDNA are vulnerable to oxidative stress damage induced by both environmental pollutants and endogenous ROS production formed in the electron transport chain of the mitochondrial matrix (Burton et al., 2017; Roubicek and Souza-Pinto, 2017). Several studies have shown that maternal exposure to ambient air pollutants is associated with reduced placental mtDNA copy number (mtDNAcn) (Janssen et al., 2012, 2015; Clemente et al., 2017). In contrast, placentas from early-onset PE had increased mtDNAcn accompanied with increased mitochondrial mass when compared to normal pregnancies, whereas there was no difference between mtDNAcn in late-onset PE and control placentas (Vishnyakova et al., 2016). Moreover, placentas from pregnancies complicated by PE have shown mitochondrial swelling and broken cristae along with excess endogenous production of ROS (Muralimanoharan et al., 2012).

Here, we hypothesize that exposure to ambient air pollution during pregnancy induces placental dysfunction seen in PE, measured as shorter telomere length and lower mtDNAcn. The aim of this study was therefore to explore the association between prenatal exposure to ambient NO_x_ and PE, as well as potential mediation effects of placental telomere length and mtDNAcn.

## Materials and Methods

### Study Design and Population

**Figure 1:**
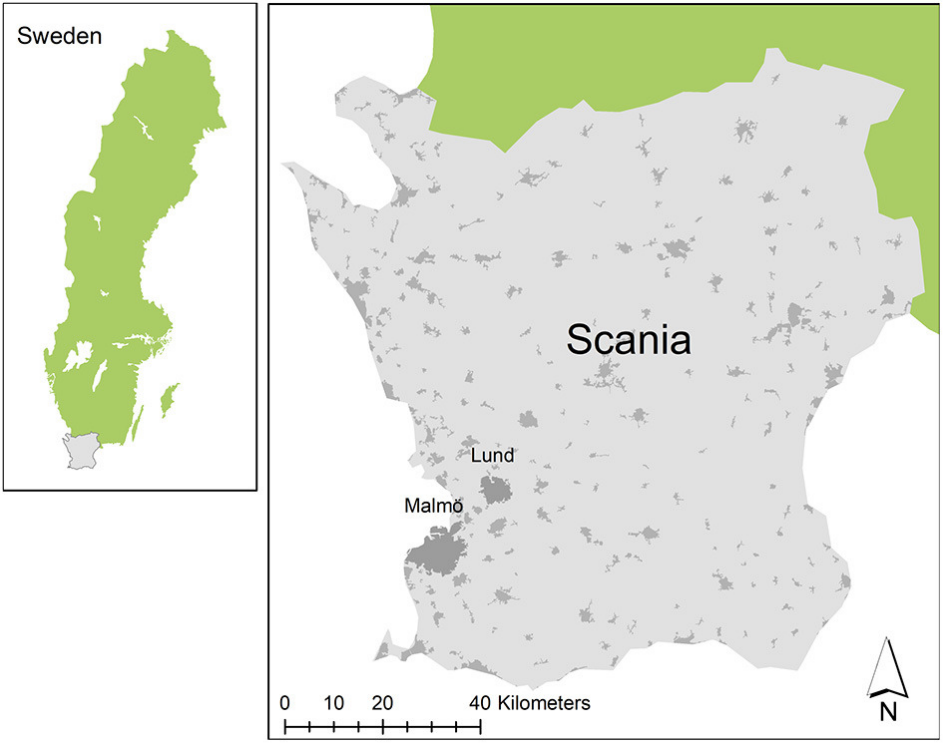
Map of study setting in Scania, Sweden.

This cross-sectional study used biobank data of placental biopsies collected from Cesarean and vaginal births between 2008 and 2015 from normotensive and PE pregnancies at Skåne University Hospital in Lund and Malmö, Sweden (Figure 1). The study protocol of placental sample collection (Dnr 243/2005) and air pollution exposure assessment at geocoded residential locations of participants (Dnr 696/2014) were approved by Lund University Ethical Review Board, LU803-2 (2015/14) and a written informed consent was obtained from all participants.

PE is recorded in the Swedish Medical Birth Register in accordance with the Swedish adaptation of the 10th version of the International Statistical Classification of Diseases and Related Health Problems (ICD-10) by the World Health Organization (WHO) (Socialstyrelsen, 2018). We selected all available PE cases and chose random controls arbitrarily for the present study. Using the unique personal identification numbers in Sweden, the biobank consists of a compiled database of medical information regarding prenatal visits, delivery and perinatal outcomes. As PE or no PE was the prerequisite for collection of placental samples, this data was also inserted and double-checked with data from prenatal visits.

**Figure 2:**
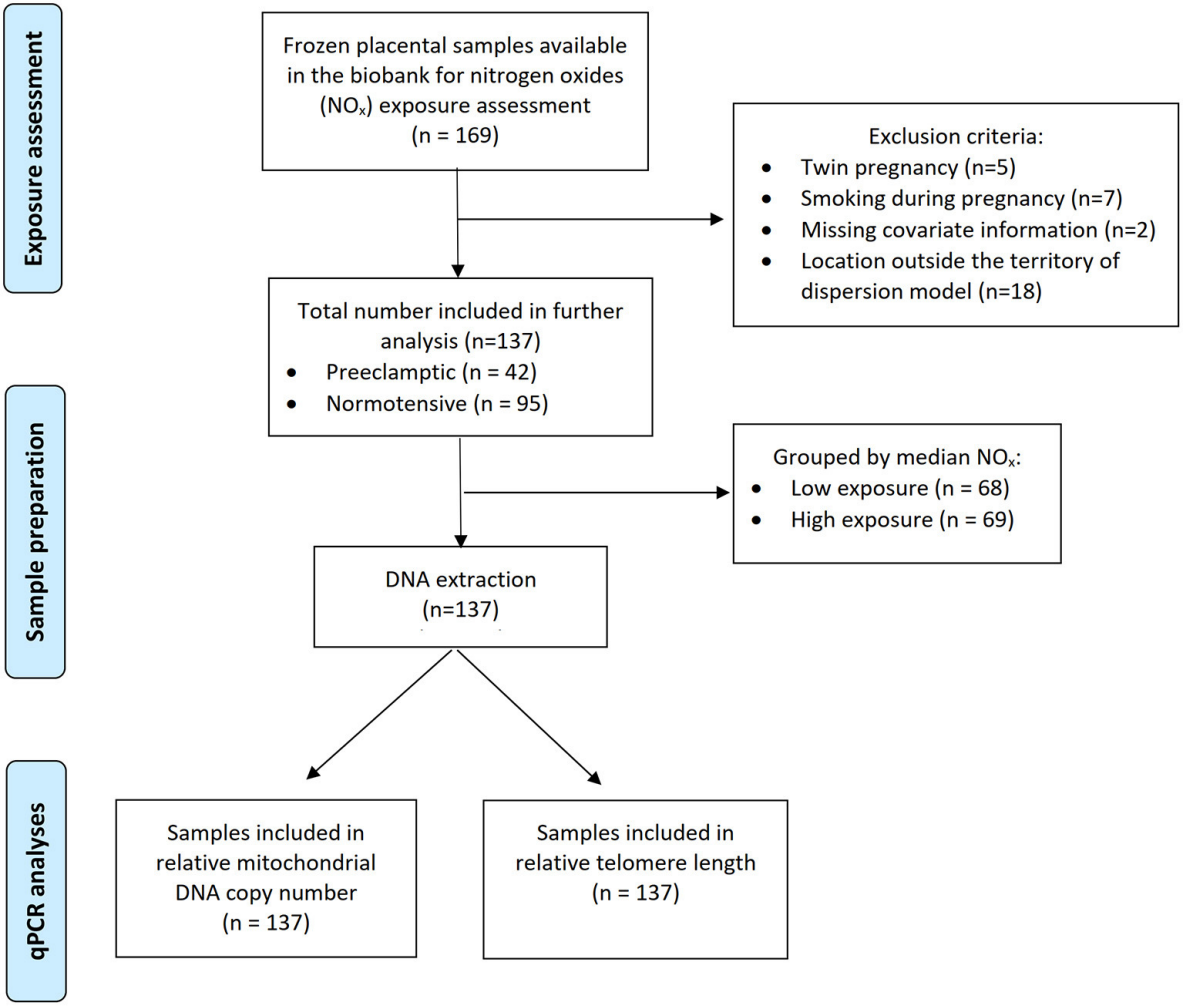
Flowchart of the study.

A flow chart of this study is shown in Figure 2. A total of 169 frozen placental tissue samples were available in the biobank for retrospective NO_x_ exposure assessment during pregnancy of the participants. It was, however, not possible to estimate the exposure for eight participants due to their residential location outside the territory of our dispersion model and they were thus excluded. In addition, we excluded participants who had twins or smoked during pregnancy. Two participants with missing covariate information were also excluded. The remaining 137 participants were included for further analysis.

### Air Pollution Exposure

Using the Swedish personal identification numbers and linking them to the regional population registry, we obtained geocoded residential locations of each participant in Scania, Sweden. Ambient concentrations of NO_x_ during pregnancy were estimated at outdoor ground level at their geocoded residential locations using EnviMan software (Opsis, 2006). The Gaussian-plume dispersion model, AERMOD developed by the United States Environmental Protection Agency (USEPA, 1998), was modified and incorporated into EnviMan. The dispersion model used in this study is explained in detail elsewhere (Rittner et al., 2020). Briefly, EnviMan relies on the emission database created by the local Air Quality Board and maintained by the Environmental Department at the City of Malmö. Sources in the emission database are road traffic, shipping, aviation, rail transport, industry, power plants, small-scale heating, machinery, as well as local emissions from Zealand, Denmark.

EnviMan calculates hourly-average concentrations of NO_x_ at a spatial resolution of 100 × 100 m from an emission rate and meteorological conditions including the wind speed, wind direction, temperature and solar radiation (Stroh et al., 2007). Our Gaussian dispersion model was only modeled for southwestern Scania. In a similar setting, Stroh et al. (2012) have found the modeled residential NO_2_ levels were strongly correlated (r_S_ = 0.8, *p* > 0.001) with measured façade NO_2_ levels. According to our previous studies, a total NO_x_ concentrations were used in the data analysis by adding a background concentration of 2.5 μg/m^3^ on the modeled hourly NO_x_ concentrations to account for transboundary air pollution (Malmqvist et al., 2013; Mandakh et al., 2020). We averaged hourly NO_x_ concentrations to monthly mean NO_x_ concentrations for each pregnant women's pregnancy period. The mean of at least 2 monthly average concentrations of NO_x_ were used as the corresponding trimester mean NO_x_ concentrations. Furthermore, third trimester mean NO_x_ concentrations were estimated based on their gestational age of delivery. In each trimester there were different number of pregnant women with complete data on exposure. In the first trimester there were 129, in the second and third trimesters there were 124 out of 137 total cases. One hundred seventeen women had complete data on exposure for all three trimesters. As our study sample size was relatively small, we used a standard method of dichotomizing a continuous variable by median to categorize statistically the trimester-specific NO_x_ concentrations to low and high exposure groups.

This study was limited to a single-pollutant analysis of ambient NO_x_ although our dispersion model had been used in the similar setting to model other ambient air pollutants, such as PM_2.5_ (particulate matter with an aerodynamic diameter smaller than 2.5 μm), PM_10_ and black carbon as reported in our population-based study (Mandakh et al., 2020). The participants given birth prior to 2009 in the present study were overlapping with Mandakh et al. (2020) and had data on locally generated PM_2.5_ and black carbon. We could not model these pollutants for those given birth after 2009 due to some missing data of PM_2.5_ measurement at local monitoring stations and economic constraint during the retrospective air pollution exposure assessment. As previously reported, modeled ambient NO_x_ was highly and positively correlated with modeled local PM_2.5_ (*r* = 0.90, *p* < 0.01) (Mandakh et al., 2020).

### Biological Sample Collection

Following a standardized protocol as recommended by Burton et al. (2014), biopsies (1 × 1 × 1 cm) from a central part of the villi region of the placenta (within a 7 cm radius of the umbilical cord) were collected within 30 min after delivery. Samples were rinsed in a sodium chloride solution, dried, and then frozen on dry ice and stored at −80°C until further analysis.

### DNA Extraction

Total DNA was extracted from the frozen placental biopsies kept on dry ice using Qiagen AllPrep DNA/RNA/Protein Mini Kit (Qiagen, Hilden, Germany) according to manufacturer's instruction, and thereafter stored at −80°C until further use. The DNA samples were thawed only once in preparation for the present study. The purity ratios [A260/280 1.87 (IQR 1.86, 1.88) and A260/230 2.05 (IQR 1.73, 2.20)] and DNA yield (ng/μl) were determined by using a NanoDrop Spectrophotometer ND-1,000 (NanoDrop technologies, Wilmington, USA).

### Quantification of Mitochondrial DNA Copy Number and Telomere Length

To quantify relative mtDNAcn and telomere length, real-time polymerase chain reactions (qPCR) were performed using an Applied Biosystems 7900HT Fast Real-Time PCR System (Applied Biosystems, Foster City, CA, USA). The PCR products of mtDNAcn and telomere length were related to PCR product of a single copy gene [hemoglobin beta (*HBB*)]. *MT-TL1* encoding the Mitochondrially Encoded TRNA-Leu (UUA/G) 1 was the target gene that was used to determine mtDNAcn (Venegas and Halberg, 2012). Master mixes for mtDNAcn quantification was prepared with PerfeCTa SYBR Green FastMix PCR reagent (ROX) (2x) (QuantaBio, Beverly, MA, USA), 10 μM mtDNA forward primer tRNA F3212 (5′-CAC CCA AGA ACA GGG TTT GT-3′), and 10 μM reverse primer tRNA R3319 (5′-TGG CCA TGG GTA TGT TGT TA-3′) (Custom DNA Oligos Synthesis Services, ThermoFisher Scientific). *HBB* master mix consisted of Fast SYBR Green Master Mix (2x) (Applied Biosystems, Waltham, USA), 10 μM *HBB* forward primer (5′-TGT GCT GGC CCA TCA CTT TG-3′), and 10 μM reverse primer (5′-ACC AGC CAC CAC TTT CTG ATA GG-3′) (Xu et al., 2018). Master mixes for telomere length runs were prepared with PCR buffer (10x) (Invitrogen, Carlsbad, CA, USA), 50 mM MgCl_2_, 10 mM dNTPs, 20 μM TEL1 primer (5'-CGG TTT GTT TGG GTT TGG GTT TGG GTT TGG GTT TGG GTT-3'), 20 μM TEL2 primer (5'-GGC TTG CCT TAC CCT TAC CCT TAC CCT TAC CCT TAC CCT-3'), 10x SYBR Green I (Invitrogen, Carlsbad, CA, USA) (50x), ROX (1x) (Invitrogen, Carlsbad, CA, USA), and 0.5 U *Taq* Platina DNA polymerase (Invitrogen, Carlsbad, CA, USA) (Herlin et al., 2019). Each reaction consisted of 2.5 μl DNA (4 ng/μl for mtDNA and *HBB*, 2 ng/μl for telomere length) and 7.5 μl master mix, resulting in total volume of 10 μl.

The thermal cycling profile of mtDNAcn PCR started with 3 min at 95°C followed by 35 cycles of 15 s of denaturation at 95°C and 1 min of annealing/extension at 60°C. *HBB* PCR condition was 20 s at 95°C, followed by 40 cycles of 1 s at 95 °C and 20 s at 60°C (Xu et al., 2018). PCR conditions for telomere length were 3 min at 95°C followed by 25 cycles of 15 s at 95°C and 1 min at 56°C (Herlin et al., 2019). The quantification of mtDNAcn, telomere length, *HBB* gene copy number and the standard curves were run in triplicates at separate runs. To generate a standard curve ranging from 0.25 to 16 ng/μl, a series of two-fold dilutions were carried out for three individual reference DNA samples. *R*^2^ of each standard curve was >0.99. Relative quantification of the concentration of mtDNAcn was determined by standard curve analysis and the ratio between mtDNAcn and *HBB* gene copy number (Xu et al., 2018). Similarly, the relative telomere length was the quotient of telomere length and *HBB* gene copy number (Herlin et al., 2019).

### Statistical Analysis

All statistical analyses were performed using the IBM SPSS Statistics software version 25 (IBM, Chicago, IL, USA). Continuous data was checked for normality and presented as means ± SD or as median with interquartile range (IQR) when data were not normally distributed. Categorical data are presented as numbers and percentage. A *p*-value of <0.05 was considered statistically significant. After NO_x_ exposure assessment, 20 subjects were missing one (*n* = 7) or two (*n* = 13) trimester-specific NO_x_ exposure data although they were term pregnancies. To account for a cumulative effect of exposure during the entire period of pregnancy, complete case analysis (*n* = 117) on complete exposure data at all trimesters was performed and reported.

For analysis of associations between prenatal NO_x_ exposure and risk of PE, adjusted odds ratios (OR) with their 95% confidence intervals (95% CI) were estimated using logistic regression, after controlling for maternal age, pregestational body mass index (BMI), parity, gestational age and fetal sex. These covariates were defined based on a priori knowledge (Pedersen et al., 2014; Clemente et al., 2017; Martens et al., 2017; Iodice et al., 2018; Mandakh et al., 2020; Melody et al., 2020). The season of birth was also chosen as a covariate to control due to the seasonal variations in both PE prevalence (Wacker et al., 1998; Phillips et al., 2004; Rudra and Williams, 2005) and NO_x_ concentrations (Pedersen et al., 2014; Choi et al., 2015; Silva et al., 2020). Linear regression was used to identify how prenatal NO_x_ exposure was associated with placental relative mtDNAcn and telomere length, respectively, after adjusting for the same set of confounders adjusted for the logistic regression model mentioned above.

The standard Baron and Kenny (1986) method of mediation analysis was employed for investigating whether placental relative mtDNAcn or telomere length mediated the association between prenatal NO_x_ exposure and PE. The Baron and Kenny method is a three step method where in the present study: (1) association between prenatal NO_x_ exposure and PE was investigated; (2) association between prenatal NO_x_ exposure and placental relative mtDNAcn or telomere length was investigated; and (3) if the association between prenatal NO_x_ exposure and placental relative mtDNAcn or telomere length was statistically significant, mtDNAcn or telomere length were entered in to the regression model of the first step (i.e., prenatal NO_x_ exposure in association with PE). In all models, the same set of confounding factors were included into the models. To be considered a mediating variable in the third step: (1) placental relative mtDNAcn or telomere length should be statistically significant; and (2) the coefficient for prenatal NO_x_ exposure should be substantially diminished as compared to in the first step.

## Results

### Characteristics

A total of 42 PE cases consisted of three early-onset PE and 39 late-onset PE. Table 1 shows characteristics of the study participants stratified by PE status. Preeclamptic women were more likely to be older than 35 years, primiparous, obese and had PE or gestational hypertension in their previous pregnancies, as compared to normotensive pregnant women. Also, they were more likely to give birth during the autumn. Aspirin and antihypertensive drug use during pregnancy was more prevalent among preeclamptic women compared with normotensive women, since aspirin had been routinely prescribed for high-risk pregnancies to reduce the risk of PE. There were no differences in placental relative telomere length and mtDNAcn between PE and normotensive pregnant women.

**Table 1:** Characteristics of study participants stratified by preeclampsia status.

**Characteristics**	**Total (*n* = 137)**	**PE (*n* = 42)**	**Normotensive (*n* = 95)**	***p*-value**
Maternal age in years [mean (SD)]	30.1 ± 4.7	30.5 ± 5.9	29.97 ± 4.1	
<35	112 (81.8)	31 (73.8)	81 (85.3)	0.110
≥35	25 (18.2)	11 (26.2)	14 (14.7)	
Parity [*n* (%)]				
Nullipara	92 (67.2)	24 (57.1)	68 (71.6)	0.228
Primipara	34 (24.8)	13 (31.0)	21 (22.1)	
Multipara	11 (8.0)	5 (11.9)	6 (6.3)	
Pregestational BMI [kg/m^3^]	25.7 ± 5.3	27.6 ± 6.3	24.9 ± 4.5	
<18.5	3 (2.2)	1 (2.4)	2 (2.1)	0.008^*^
18.5–24.9	66 (48.2)	16 (38.1)	50 (52.6)	
25–29.9	40 (29.2)	9 (21.4)	31 (32.6)	
≥30	28 (20.4)	16 (38.1)	12 (12.6)	
Fetal sex [*n* (%)]				
Male	73 (53.3)	23 (54.8)	50 (52.6)	0.818
Female	64 (46.7)	19 (45.2)	45 (47.4)	
Gestational age, days [mean (SD)]	274.2 ± 15.8	269.3 ± 14.6	276.3 ±15.9	
Birth weight [g]	3455.1 ± 702.2	3323.1 ± 748.9	3513.4 ± 676.4	
Previous PE [*n* (%)]	16 (11.7)	8 (19.0)	8 (8.4)	0.074
Previous gestational HTN^a^ [*n* (%)]	7 (5.1)	3 (7.1)	4 (4.2)	0.472
Medication of importance [*n* (%)]				
Aspirin	6 (4.4)	5 (11.9)	1 (1.1)	0.01^*^^b^
Antihypertensive drugs	19 (13.9)	16 (38.1)	3 (3.2)	<0.001^*^
Mode of delivery [*n* (%)]				
Vaginal	106 (79.1)	31 (75.6)	75 (80.6)	0.515
Cesarean	28 (20.9)	10 (24.4)	18 (19.4)	
Municipality [*n* (%)]				
Malmö	35 (25.9)	8 (20.0)	27 (28.4)	0.505
Lund	46 (34.1)	15 (37.5)	31 (32.6)	
Other	54 (40.0)	17 (42.5)	37 (38.9)	
Season of birth [*n* (%)]				
Winter	18 (13.1)	6 (14.3)	12 (12.6)	0.055
Spring	26 (19.0)	4 (9.5)	22 (23.2)	
Summer	39 (28.5)	9 (21.4)	30 (31.6)	
Autumn	54 (39.4)	23 (54.8)	31 (32.6)	
Placental mtDNAcn				
Mean ± SD	0.88 ± 0.39	0.87 ± 0.40	0.88 ± 0.39	
Median (IQR)	0.82 (0.57–1.08)	0.82 (0.49–1.14)	0.83 (0.59–1.05)	
Placental telomere length				
Mean ± SD	1.39 ± 0.36	1.36 ± 0.33	1.41 ± 0.38	
Median (IQR)	1.36 (1.16–1.62)	1.35 (1.12–1.58)	1.37 (1.16–1.63)	

* *Pearson Chi-Square p < 0.05*;

a *HTN, Hypertension*;

b *Fisher's Exact test*.

**Figure 3:**
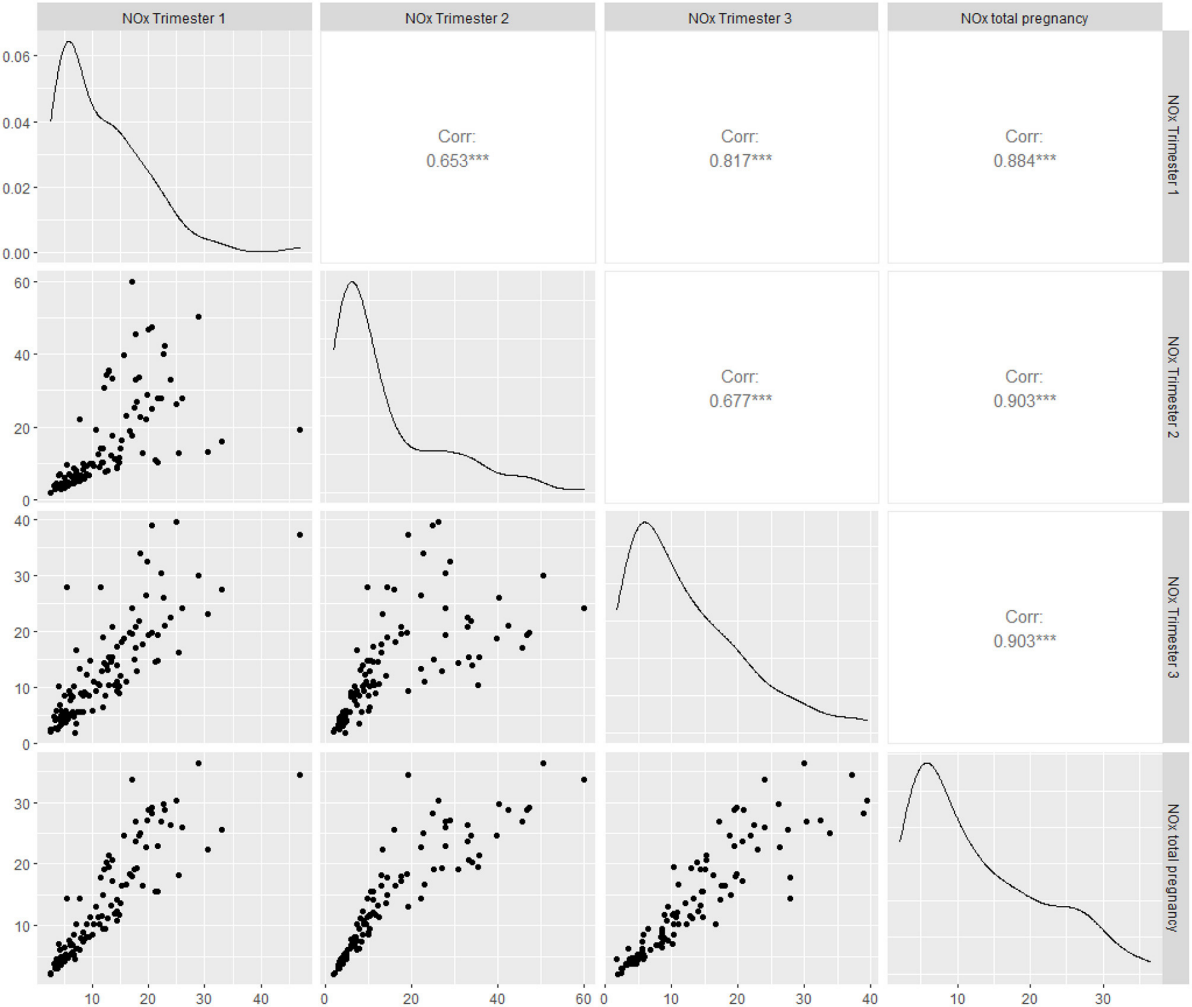
Correlation plot of NO_x_ concentrations at all trimesters and entire period of pregnancy.

The mean NO_x_ concentrations varied between 14.0 and 16.2 μg/m^3^ over the three trimesters and entire period of pregnancy (Table 2). As shown in Figure 3, the trimester-specific and total NO_x_ concentrations had moderate (r_p_ = 0.65, *p* < 0.001) to high (r_p_ = 0.90, *p* < 0.001) correlations that were statistically significant. The range in ambient NO_x_ was wide, modeled NO_x_ concentrations were split to low and high exposure groups by median cut-offs during first (11.9 μg/m^3^; IQR: 7.9, 17.9), second (11.6 μg/m^3^; IQR: 7.1, 21.1), third trimesters (11.9 μg/m^3^; IQR: 7.7, 19.5), and entire period of pregnancy (12.0 μg/m^3^; IQR: 7.6, 20.1) (Table 2). The characteristics of study population stratified by ambient NO_x_ exposure group is presented in the Supplementary Table 1. Those exposed to high NO_x_ concentrations during pregnancy were more likely to be primipara, overweight and to give birth in the spring and summer.

**Table 2:** Descriptive statistics of prenatal NO_x_ exposure (μg/m^3^).

**Time window**	***N***	**Mean ± SD**	**Min**	**P25**	**P50**	**P75**	**Max**
Trimester 1	129	14.0 ± 7.6	5.1	7.9	11.9	17.9	49.5
Trimester 2	124	16.2 ± 12.4	4.4	7.1	11.6	21.1	62.5
Trimester 3	124	14.7 ± 8.6	4.3	7.7	11.9	19.5	41.9
Entire pregnancy	137	14.8 ± 8.4	4.6	7.6	12.0	20.1	38.9

### Prenatal NO_x_ Exposure and PE

Independent of maternal age, pregestational BMI, parity, season of birth and fetal sex, statistically significant associations were found between prenatal NO_x_ exposure and PE when looking at exposure during first trimester (OR 4.0; 95% CI: 1.4, 11.1; *p* = 0.008) and entire pregnancy (OR 3.7; 95% CI: 1.3, 10.4; *p* = 0.012) (Table 3). Effect estimates associated with NO_x_ exposures during the second and third trimesters were decreased, but statistically not significant.

**Table 3:** Odds ratios (OR) and their 95% Confidence Intervals (CI) for PE in association with high NO_x_ exposure as compared with low NO_x_ exposure (median cut-off).

**Time window**	**Exposure group**	***B***	**Adjusted OR** ^**§**^	**95% CI for AOR**		***p***
				**Lower**	**Upper**	
First trimester	Low	Reference				
	High	1.38	4.0	1.4	11.1	0.008
Second trimester	Low	Reference				
	High	1.02	2.8	0.97	8.0	0.056
Third trimester	Low	Reference				
	High	0.91	2.5	0.93	6.7	0.071
Entire pregnancy	Low	Reference				
	High	1.31	3.7	1.3	10.4	0.012

§ *Adjusted for maternal age, pregestational BMI, parity, gestational age, season of birth and fetal sex*.

### Prenatal NO_x_ Exposure, Placental Relative mtDNAcn and PE

Women exposed to high concentrations of NO_x_ had lower placental mtDNAcn (−0.20, 95% CI: −0.36, −0.04; *p* = 0.01) as compared with women exposed to low NO_x_ concentrations during the first trimester (Table 4). The association between prenatal NO_x_ exposure and placental relative mtDNAcn during other time windows were in the same direction, but the effect estimates were slightly lower and not statistically significant. Placental mtDNAcn was not associated with PE neither in unadjusted (OR 0.8; 95% CI: 0.3, 2.1; *p* = 0.59) nor in adjusted analyses (OR 0.6; 95% CI: 0.2, 2.1; *p* = 0.46).

**Table 4:** Linear regression analysis of placental relative mtDNAcn in women with high NO_x_ exposure as compared with women with low NO_x_ exposure (median cut-off).

**Time window**	**Exposure group**	**Unadjusted change in mtDNAcn, (95% CI)**	***p***	**Adjusted**^**§**^ **change in mtDNAcn, (95% CI)**	***p***
First trimester	Low	Reference		Reference	
	High	−0.17 (−0.31, −0.03)	0.02	−0.20 (−0.36, −0.04)	0.01
Second trimester	Low	Reference		Reference	
	High	−0.15 (−0.30, −0.01)	0.04	−0.16 (−0.33, 0.01)	0.06
Third trimester	Low	Reference		Reference	
	High	−0.16 (−0.30, −0.01)	0.03	−0.15 (−0.31, 0.01)	0.06
Entire pregnancy	Low	Reference		Reference	
	High	−0.13 (−0.27, 0.02)	0.09	−0.14 (−0.31, −0.02)	0.08

§ *Adjusted for maternal age, pregestational BMI, parity, gestational age, season of birth and fetal sex*.

### Mediation Analysis

There was an association between prenatal NO_x_ exposure during first trimester and PE, as well as an association between prenatal NO_x_ exposure during first trimester and placental relative mtDNAcn. To investigate if placental mtDNAcn was mediating this association, we performed a mediation analysis.

In Table 5, *Y* is the dependent variable, i.e., PE; *X* the independent variable, prenatal NO_x_ exposure; and *Me* the proposed mediator variable, mtDNAcn. In order for a variable to be considered a mediator, β_32_ should be statistically significant, however this was not the case (*p* = 0.954). Furthermore, β_31_ should be either substantially smaller than *β*_11_, which it was slightly smaller (*β*_11_ = 1.382 > *β*_31_ = 1.375), or statistically not significant, which it was not (*p* = 0.010). Therefore, mtDNAcn was not a mediator for the association between prenatal NO_x_ exposure and PE (Table 5).

**Table 5:** Mediation analysis to determine the causal mediated effect of placental relative mtDNAcn on the association between prenatal NO_x_ exposure and PE.

	***Model***	***β***_**i**_	***p*-value (s)**
Step 1	*Y* = *β*_10_ + *β*_11_*X* + *ε*_1_	*β*_11_ = 1.382	0.008
Step 2	*Me* = *β*_20_ + *β*_21_*X* + *ε*_2_	β_21_ = −0.201	0.014
Step 3	*Y* = *β*_30_ + *β*_31_*X* + *β*_32_*Me* + *ε*_3_	*β*_31_ = 1.375 *β*_32_ = −0.037	0.010 0.954

*β_i_, b-coefficients*;

*ε_i_, standard errors*.

### Prenatal NO_x_ Exposure, Placental Relative Telomere Length and PE

There was no statistically significant difference in placental relative telomere length between exposure groups (Table 6). There was no significant association between placental relative telomere length and PE neither in unadjusted (OR 0.6; 95% CI: 0.2, 2.0; *p* = 0.43) nor in the adjusted analyses (OR 0.4; 95% CI: 0.1, 1.4; *p* = 0.14). Since the assumption of mediation analysis did not meet, we did not further investigate any mediation effect of placental relative telomere length on the association between prenatal NO_x_ exposure and PE.

**Table 6:** Linear regression analysis of placental telomere length in women with high NO_x_ exposure as compared with women with low NO_x_ exposure (median cut-off).

**Time window**	**Exposure group**	**Unadjusted change in placental relative telomere length (95% CI)**	***p***	**Adjusted**^**§**^ **change in placental relative telomere length (95% CI)**	***p***
First trimester	Low	Reference		Reference	
	High	0.01 (−0.11, 0.14)	0.82	0.03 (−0.11, 0.17)	0.67
Second trimester	Low	Reference		Reference	
	High	0.04 (−0.08, 0.17)	0.51	0.07 (−0.08, 0.21)	0.37
Third trimester	Low	Reference		Reference	
	High	0.04 (−0.09, 0.16)	0.57	0.07 (−0.06, 0.21)	0.30
Entire pregnancy	Low	Reference		Reference	
	High	0.06 (−0.07, 0.18)	0.37	0.08 (−0.06, 0.21)	0.27

§ *Adjusted for maternal age, pregestational BMI, parity, gestational age, season of birth and fetal sex*.

## Discussion

We found that prenatal exposure to ambient NO_x_ during the first trimester was associated with reduced placental relative mtDNAcn and increased risk of PE, which indicate that early pregnancy is the most vulnerable period for damage of the placenta by ambient air pollution. In contrary to our hypothesis, we found no evidence that placental relative mtDNAcn mediate the association between ambient NO_x_ and PE. Placental relative telomere length was not associated with prenatal NO_x_ exposure or with PE.

Early pregnancy exposure to ambient NO_x_ coincides with epigenetic reprogramming of germ cells and preimplantation embryos by DNA methylation occurring in the first trimester (Reik et al., 2001). A plausible mechanism linking prenatal NO_x_ exposure and PE may be abnormal trophoblast fusion, which is a defining characteristic of PE. The maternal-fetal interface of a placenta consists of syncytiotrophoblasts that are formed through trophoblast fusion (Roland et al., 2016). Normal trophoblast syncytialization requires fully functional mitochondria (Poidatz et al., 2015). However, reduced placental mtDNAcn due to prenatal NO_x_ exposure reflects mitochondrial dysfunction which may interfere with trophoblast fusion (Walker et al., 2020). Although the exact mechanisms remain to be clarified, the reduced placental mtDNAcn in association with higher exposure to NO_x_ in the present study could give an indication that air pollution may be a contributing factor for dysfunctional mitochondrial biogenesis during pregnancy. As previously shown, exposure to ambient PM during pregnancy has been found to be associated with increased levels of mitochondrial 8-hydroxy 2′-deoxyguanine, which is considered to be a marker of oxidative DNA damage (Grevendonk et al., 2016) which was found to be correlated with loss of mtDNA (Qian and Van Houten, 2010). Taken together, these findings suggest that ambient NO_x_ exposure during first trimester may play a role in placental dysfunction seen in PE.

However, mediation analysis of placental mtDNAcn undertaken here, did not appear to influence the relationship between prenatal NO_x_ exposure and PE. Interestingly, the only previous study analyzing mtDNAcn as a mediator found that 10% of the association between maternal exposure to nitrogen dioxide (NO_2_) during pregnancy and birth weight, was mediated by a decrease in placental mtDNAcn (Clemente et al., 2016). The sample size was larger (*n* = 926) in that study and the mean NO_2_ concentrations ranged between 21.1 and 25.5 μg/m^3^ which is higher than the mean NO_x_ concentrations (14–16.2 μg/m^3^) in our study, which could possibly explain the effect of mediation. Future research in a larger cohort is therefore needed to assess the value of this biomarker for assessing mitochondrial dysfunction in placental pathology.

Although direct deleterious effects of ambient NO_x_ on placenta have not previously been studied, the detrimental effect of fine and ultrafine particles on placenta have been reported. Ambient black carbon particles have been found to be translocated to the fetal side of the placenta collected from full term pregnancies (Bové et al., 2019). A recent *in-vitro* study of first trimester trophoblast cells exposed to PM_2.5_ indicated morphological changes, accompanied by altered protease and H_2_O_2_ production in mitochondria (Nääv et al., 2020). In another recent *in-vitro* study, exogenous NO_x_ has been found in the placental homogenates from healthy term pregnancies in the form of iron nitrosyl complexes (Mukosera et al., 2020). Furthermore, placental villous tissue collected from women with pregnancies complicated by PE-FGR had increased levels of NO_x_ as compared to normotensive pregnancies with FGR (Mukosera et al., 2020). Whether prenatal exposure to NO_x_ leads to placental mitochondrial dysfunction, irrespective of the simultaneous effect of PM, remains to be determined in future studies.

In the present study, the effect of prenatal exposure to PM on placental mtDNAcn or PE was not evaluated. Both NO_x_ and PM mainly, however, originate from various combustion processes, such as a combustion engine. In our previous population-based study in southern Scania, there was a statistically significant, high positive correlation between mean NO_x_ and local PM_2.5_ concentrations (r_p_ = 0.90, *p* < 0.01) and mean NO_x_ and total PM_2.5_ concentrations (r_p_ = 0.58, *p* < 0.01) during the entire pregnancy (Mandakh et al., 2020). Therefore, it can be hypothesized that exposure to NO_x_ during pregnancy is representative also for exposure to locally emitted PM_2.5_, however, given the difference in biological fate of PM_2.5_ and NO_x_ in human body, it is not possible to account the change in placental mtDNAcn observed in the NO_x_ exposure model to PM_2.5_. Nevertheless, this study do not have the data to fully support this hypothesis since we could only model NO_x_ exposure for the majority of participants.

In contrast to earlier findings, however, we found no association between prenatal NO_x_ exposure and placental telomere length, nor between placental telomere length and PE. Previous studies reported associations between telomere shortening in placenta and nearest proximity to major roads (Bijnens et al., 2015) and with maternal exposure to PM_2.5_ during pregnancy (Martens et al., 2017). A 5 μg/m^3^ increment in PM_2.5_ exposure during the entire pregnancy was associated with 13.2% decrease in placental telomere length (Martens et al., 2017). As compared to our study, Martens et al. (2017) had a larger sample size (*n* = 641) and a larger variation (4.3–32.5 μg/m^3^) in weekly PM_2.5_ exposure. Furthermore, shorter telomeres were found in placental trophoblasts from pregnancies complicated by PE, FGR and PE with FGR as compared to uncomplicated pregnancies (Biron-Shental et al., 2010). However, the finding that telomere shortening was not found in cord blood cells from pregnancies complicated with PE suggests that air pollution-related shorter telomere length might be restricted to placental trophoblasts (Sukenik-Halevy et al., 2016).

To our knowledge, this is the first study to report an association between prenatal exposure to ambient NO_x_, placental dysfunction measured with biomarkers of toxicity, and PE. Another key strength of this study is the access to a unique biobank specially created for studying preeclampsia with extensive biological samples linked with detailed clinical data. In addition, the individual exposure was assessed at residence level by high resolution dispersion model after obtaining the geocoded residential locations of each participant by linking the unique personal identification number with the well-documented regional population registry. Further studies, which take these biomarkers of toxicity into account, should also measure oxidative stress biomarkers in the placenta to better explain the association between prenatal ambient air pollution exposure and the risk of PE.

The generalizability of these results is subject to at least four limitations. Firstly, our study might be predisposed to exposure misclassification due to a residual confounder related to unmeasured socioeconomic status of the participants and not accounting for exposure during commuting, work, or indoor exposure. We were not able to obtain data on building characteristics and socioeconomic status of the participants. Difficulties to assess the total exposure is, however, a common limitation in air pollution epidemiologic studies (Pedersen et al., 2014). Secondly, it is beyond the scope of this study to further examine associations with sub-clinical diagnosis of PE such as early- and late-onset PE, or PE with or without FGR, due to the limited sample size. In addition, we were not able to match PE cases with normotensive controls based on covariates such as gestational age due to the fact that they were not equally distributed among the term and preterm births after the exposure assessment. Moreover, we could not exclude the fact that prematurity can be a result of other pathologic pregnancies and therefore a preterm normotensive pregnant women were not selected as a preterm control to match preterm PE cases. Lastly, the current study has only examined NO_x_ exposure due to the technical error and economic constraint to model other ambient air pollutants, such as PM_2.5_ during exposure assessment.

Our data show that exposure to ambient air pollution (NO_x_) during the first trimester was associated with reduced placental relative mtDNAcn and an increased risk of PE. However, we did not find any evidence that mtDNAcn or TL mediated the association between air pollution and PE. Future research should further investigate the role of mtDNAcn for pregnancy complications in relation to exposure to ambient air pollution during pregnancy.

## Data Availability Statement

The datasets related to the clinical parameters and the DNA-analysis are all available upon request. However the dataset related to individual exposure levels of air pollution are not readily available because of limitations set in the Ethical permission stating that only the researchers involved in the project are allowed to access data. Requests to access the dataset should be directed to data-holders; Statistic Sweden and The Swedish National Board of Health and Welfare for birth registry data.

## Ethics Statement

The studies involving human participants were reviewed and approved by Lund University Ethical Review Board, LU803-2 (2015/14). The patients/participants provided their written informed consent to participate in this study.

## Author Contributions

EM, YM, SH, KB, AO, CI, and LE: conceptualization, methodology, and writing—review and editing. YM, KB, and AO: formal analysis and investigation. KB, SH, and LE: resources. EM, SH, and LE: data curation. YM: writing—original draft preparation. EM, AO, KB, and SH: supervision. EM: project administration. EM, CI, and SH: funding acquisition. All authors have read and approved the submitted version of the manuscript.

## Conflict of Interest

The authors declare that the research was conducted in the absence of any commercial or financial relationships that could be construed as a potential conflict of interest.
