# Supplementary material for: Association of Prenatal Ambient Air Pollution Exposure With Placental Mitochondrial DNA Copy Number, Telomere Length and Preeclampsia
Source: Front Toxicol. 2021 May 26;3:659407. doi: 10.3389/ftox.2021.659407 (PMC8915808; doi:10.3389/ftox.2021.659407)
Supplement: Supplementary file 1 [file Table_1.DOCX]

Supplementary table 1.Characteristics of study participants stratified by ambient NO_x_ exposure groups during pregnancy.

| Characteristics | First trimester | | Second trimester | | Third trimester | | Entire pregnancy | |
| --- | --- | --- | --- | --- | --- | --- | --- | --- |
|  | Low  (n=58) | High  (n=58) | Low  (n=58) | High  (n=58) | Low  (n=58) | High  (n=58) | Low  (n=58) | High  (n=58) |
| Maternal age in years [mean (SD)] | 30.3 ± 4.9 | 30.7 ± 4.3 | 29.6 ± 4.6 | 31.4 ± 4.5 | 30.2 ± 4.5 | 30.8 ± 4.7 | 30.1 ± 4.6 | 30.8 ± 4.7 |
| <35 | 46 (79.3) | 47 (81.0) | 49 (84.5) | 44 (75.9) | 48 (82.8) | 45 (77.6) | 47 (81.0) | 46 (79.3) |
| ≥35 | 12 (20.7) | 11 (19.0) | 9 (15.5) | 14 (24.1) | 10 (17.2) | 13 (22.4) | 11 (19.0) | 12 (20.7) |
| Parity [n (%)] |  |  |  |  |  |  |  |  |
| Nullipara | 44 (75.9)* | 31 (53.4)* | 46 (79.3)* | 29 (50.0)* | 43 (74.1) | 32 (55.2) | 42 (72.4) | 33 (56.9) |
| Primipara | 11 (19.0)* | 21 (36.2)* | 10 (17.2)* | 22 (37.9)* | 13 (22.4) | 19 (32.8) | 13 (22.4) | 19 (32.8) |
| Multipara | 3 (5.2)* | 6 (10.3)* | 2 (3.4)* | 7 (12.1)* | 2 (3.4) | 7 (12.1) | 3 (5.2) | 6 (10.3) |
| Pregestational BMI [kg/m^3^] | 26.7 ± 6.0 | 25.2 ± 4.8 | 26.8 ± 6.1 | 25.1 ± 4.7 | 27.0 ± 6.1 | 24.9 ± 4.6 | 26.7 ± 6.0 | 25.2 ± 4.8 |
| <18.5 | 1 (1.7) | 1 (1.7) | 1 (1.7) | 1 (1.7) | 0 (0.0) | 2 (3.4) | 0 (0.0) | 2 (3.4) |
| 18.5-24.9 | 27 (46.6) | 30 (51.7) | 26 (44.8) | 31 (53.4) | 27 (46.6) | 30 (51.7) | 28 (48.3) | 29 (50.0) |
| 25-29.9 | 14 (24.1) | 17 (29.3) | 15 (25.9) | 16 (27.6) | 14 (24.1) | 17 (29.3) | 15 (25.9) | 16 (27.6) |
| ≥30 | 16 (27.6) | 10 (17.2) | 16 (27.6) | 10 (17.2) | 17 (29.3) | 9 (15.5) | 15 (25.9) | 11 (19.0) |
| Fetal sex [n (%)] |  |  |  |  |  |  |  |  |
| Male | 31 (53.4) | 31 (53.4) | 31 (53.4) | 31 (53.4) | 32 (55.2) | 30 (51.7) | 29 (50.0) | 33 (56.9) |
| Female | 27 (46.6) | 27 (46.6) | 27 (46.6) | 27 (46.6) | 26 (44.8) | 28 (48.3) | 29 (50.0) | 25 (43.1) |
| Gestational age, days [mean (SD)] | 274.2 ± 15.1 | 274.4 ± 14.1 | 276.4 ± 12.6 | 272.2 ± 16.0 | 276.3 ± 12.6 | 272.3 ± 16.1 | 275.6 ± 13.1 | 273.1 ± 15.8 |
| Birth weight [g] | 3405.0 ± 654.4 | 3481.5 ± 703.6 | 3505.1 ± 564.7 | 3381.3 ± 774.3 | 3497.3 ± 586.6 | 3389.2 ± 759.1 | 3456.0 ± 594.1 | 3430.1 ± 756.9 |
| Previous PE [n (%)] | 8 (13.8) | 7 (12.1) | 8 (13.8) | 7 (12.1) | 9 (15.5) | 6 (10.3) | 7 (12.1) | 8 (13.8) |
| Previous gestational HTN^a^ [n (%)] | 2 (3.4) | 3 (5.2) | 3 (5.2) | 2 (3.4) | 3 (5.2) | 2 (3.4) | 4 (6.9) | 1 (1.7) |
| Medication of importance [n (%)] |  |  |  |  |  |  |  |  |
| Aspirin | 1 (1.7) | 4 (6.9) | 1 (1.7) | 4 (6.9) | 1 (1.7) | 4 (6.9) | 1 (1.7) | 4 (6.9) |
| Antihypertensive drugs | 5 (8.6) | 10 (17.2) | 4 (6.9) | 11 (19.0) | 3 (5.2) | 12 (20.7) | 4 (6.9) | 11 (19.0) |
| Mode of delivery  [n (%)] |  |  |  |  |  |  |  |  |
| Vaginal | 41 (70.7) | 49 (84.5) | 44 (75.9) | 46 (79.3) | 43 (74.1) | 47 (81.0) | 42 (72.4) | 48 (82.8) |
| Cesarean | 17 (29.3) | 9 (15.5) | 14 (24.1) | 12 (20.7) | 15 (25.9) | 11 (19.0) | 16 (27.6) | 10 (17.2) |
| Municipality [n (%)] |  |  |  |  |  |  |  |  |
| Malmö | 1 (1.7)* | 33 (56.9)* | 0 (0.0)* | 34 (58.6)* | 0 (0.0)* | 34 (58.6)* | 0 (0)* | 34 (58.6)* |
| Lund | 28 (48.3)* | 10 (17.2)* | 30 (51.7)* | 8 (13.8)* | 31 (53.4)* | 7 (12.1)* | 31 (53.4)* | 7 (12.1)* |
| Other | 29 (50.0)* | 15 (25.9)* | 28 (48.3)* | 16 (27.6)* | 27 (46.6)* | 17 (29.3)* | 27 (46.6)* | 17 (29.3)* |
| Season of birth [n (%)] |  |  |  |  |  |  |  |  |
| Winter | 10 (17.2)* | 7 (12.1)* | 9 (15.5)* | 8 (13.8)* | 11 (19.0)* | 6 (10.3)* | 10 (17.2)* | 7 (12.1)* |
| Spring | 4 (6.9)* | 12 (20.7)* | 3 (5.2)* | 13 (22.4)* | 3 (5.2)* | 13 (22.4)* | 3 (5.2)* | 13 (22.4)* |
| Summer | 10 (17.2)* | 26 (44.8)* | 11 (19.0)* | 25 (43.1)* | 12 (20.7)* | 24 (41.4)* | 12 (20.7)* | 24 (41.4)* |
| Autumn | 34 (58.6)* | 13 (22.4)* | 35 (60.3)* | 12 (20.7)* | 32 (55.2)* | 15 (25.9)* | 33 (56.9)* | 14 (24.1)* |
| Placental mtDNAcn |  |  |  |  |  |  |  |  |
| mean ± SD | 0.95 ± 0.43 | 0.78 ± 0.34 | 0.95 ± 0.46 | 0.78 ± 0.32 | 0.94 ± 0.44 | 0.79 ± 0.33 | 0.95 ± 0.45 | 0.78 ± 0.31 |
| median (IQR) | 0.90  (058, 1.24) | 0.74  (0.49, 1.00) | 0.90  (0.56, 1.26) | 0.75  (0.56, 0.98) | 0.87  (0.57, 1.26) | 0.75  (0.56, 0.98) | 0.87  (0.56, 1.31) | 0.75  (0.56, 0.98) |
| Placental telomere length |  |  |  |  |  |  |  |  |
| mean ± SD | 1.39 ± 0.32 | 1.41 ± 0.38 | 1.36 ± 0.32 | 1.39 ± 0.34 | 1.36 ± 0.31 | 1.39 ± 0.35 | 1.36 ± 0.31 | 1.39 ± 0.35 |
| median (IQR) | 1.37  (1.20, 1.56) | 1.35  (1.15, 1.60) | 1.35  (1.19, 1.55) | 1.36  (1.15, 1.61) | 1.35  (1.19, 1.55) | 1.36  (1.15, 1.62) | 1.35  (1.19, 1.55) | 1.36  (1.15, 1.61) |

*Pearson Chi-Square p<0.05; ^a^HTN – Hypertension; ^b^Fisher’s Exact test
